# Composition and Antioxidant Activity of Phenolic Compounds in Fruit of the Genus *Rosa* L.

**DOI:** 10.3390/antiox10040545

**Published:** 2021-04-01

**Authors:** Mindaugas Liaudanskas, Irena Noreikienė, Kristina Zymonė, Rugilė Juodytė, Vaidotas Žvikas, Valdimaras Janulis

**Affiliations:** 1 Department of Pharmacognosy, Lithuanian University of Health Sciences, Sukilėlių av. 13, LT-50162 Kaunas, Lithuania; farmakog2@lsmuni.lt (R.J.); valdimaras.janulis@lsmuni.lt (V.J.); 2 Institute of Pharmaceutical Technologies, Lithuanian University of Health Sciences, Sukilėlių av. 13, LT-50162 Kaunas, Lithuania; kristina.zymone@lsmuni.lt (K.Z.); vaidotas.zvikas@lsmuni.lt (V.Ž.); 3 Botanical Garden of Vilnius University, Kairėnų str. 43, LT-10239 Vilnius, Lithuania; irena.noreikiene@bs.vu.lt

**Keywords:** phenolic compounds, antioxidant activity, *Rosa* L. fruit, UHPLC–ESI–MS/MS analysis, UV–Vis spectrophotometry

## Abstract

We investigated the qualitative and quantitative composition of phenolic compounds in the fruit of *Rosa* L. cultivars grown in Lithuania. The highest total content of phenolic compounds (50.13 ± 4.17 mg GAE/g, *p* < 0.05) was determined in fruit samples of *Rosa pimpinellifolia* L. cultivar “Single Cherry”. The highest levels of hydroxycinnamic acid derivatives were determined in fruit samples of *Rosa rugosa* Thunb. cultivars “Dart’s Defender” and “Adam Chodun”. The highest flavonoid content was determined in fruit samples of *Rosa multiflora* Thunb. cultivar “Nana” and *R. multiflora* species. The strongest antioxidant activity evaluated by applying DPPH and FRAP assays was determined in fruit extracts of *R. pimpinellifolia* cultivar “Single Cherry” and *R. rugosa* cultivar “Adam Chodun”. Qualitative and quantitative analysis of phenolic compounds in *Rosa* L. fruit was performed by applying UHPLC. The following phenolic compounds were identified in fruit samples: caffeic acid, chlorogenic acid, quercetin, quercitrin, (+)-catechin, (−)-epicatechin, (−)-epicatechin gallate, rutin, phloridzin, and kaempferol-3-O-glycoside. A strong correlation was determined between the total amount of phenolic compounds determined in extracts of the fruit samples of *Rosa* L. cultivars and the radical scavenging and reducing activity of their extracts in vitro (*R* = 0.767 and 0.727, respectively, *p* < 0.05).

## 1. Introduction

*Rosaceae* Juss. is a family of Angiospermae consisting of about 100 genera and 2830–3100 different plant species [1]. The genus *Rosa* L. includes about 200 species that grow in natural habitats and/or are cultivated [2,3]. Only eight naturally growing species are determined in Lithuania. Most commonly, botanical raw material of the fruit is collected from *Rosa rugosa* Thunb., *Rosa majalis* Herrm., and *Rosa canina* L. plants. *Rosa* L. is widespread throughout Europe, Asia, North America, and the Middle East [4].

The fruits of *Rosa* L. (*Rosae pseudo-fructus*) have long been used as raw material for making medicinal products [5]. The fruits have been determined to contain many different groups of biologically active compounds with a wide range of biological effects. This is the reason for the wide application of this raw material and its preparations in practical medicine and food industry of Lithuania and other countries of the world [6,7,8]. The fruits have been determined to have an anti-inflammatory [9,10], antioxidant [8,11], and antiproliferative [12] effects. Clinical studies have shown that the raw material of the fruit in dietary supplements reduces the symptoms of osteoarthritis [12,13,14]. *R. canina* fruit preparations protect the kidneys from oxidative stress [15] and have anti-inflammatory, antidiabetic, antimicrobial, and anticancer effects [6].

The fruit of *Rosa* L. is widely used in food preparation. In the past, this botanical raw material was used for the preparation of beverages, jellies, and jams. Studies are underway to replace some food additives partially or completely by using *Rosa* L. fruit [16]. Recently, the raw material of the fruit has been used in the production of probiotic beverages and yogurts, as well as an ingredient in soups [17,18]. A scientific study on the effect of purified *R. pimpinellifolia* fruit extract on yogurt quality was carried out [19].

It is important to investigate the variability of the qualitative and quantitative composition of raw medicinal plant materials that can be used as new botanical food additives. The results of research on the variability of phenolic compounds in *Rosa* L. fruit described in the scientific literature are fragmentary. So far, no studies on the quantitative and qualitative composition of phenolic compounds in the fruit of *Rosa* L. growing and cultivated in Lithuania have been performed.

It is important to study the effects of extracts of *Rosa* L. fruit samples in vitro, including the antioxidant effects of phenolic compounds, which have an impact on the prevention of various diseases. The relationship between food and health is becoming increasingly important as consumers want to eat healthy, tasty, and natural food grown in an organic environment, thus maintaining a healthy and balanced diet. The scientific literature describes the results of epidemiological and clinical studies that demonstrate the impact of diet on health. This has led researchers around the world to propose new functional foods that are not only a source of nutrients but are also important for the prevention of cancer, cardiovascular disorders, and diabetes; for strengthening of the immune system; and for slowing down the aging process [20,21,22].

A broad variety of analytical methods have been employed for the detection of phenolic compounds in raw medicinal plant materials and their products. Thin-layer and high-performance thin-layer chromatography; mass, infrared, and ultraviolet spectroscopy; and other analytical methods have been applied for the qualitative analysis of phenolics [23,24]. For the quantitative evaluation of phenolic compounds in plant matrices, HPLC and UV–Vis absorption spectrophotometry are the most widely used methods, while capillary zone electrophoresis is applied less frequently [25].

Spectrophotometry is often used for the evaluation of the total amount of phenolic compounds in samples of raw medicinal plant materials. One of the disadvantages of applying UV–Vis spectrophotometry is that it does not allow for determining the qualitative and quantitative composition of individual biologically active compounds—only the total amount of phenolic compounds or their individual groups (flavonoids, hydroxycinnamic acid derivatives, etc.) can be evaluated. Plant extracts are multicomponent matrices composed of biologically active compounds of different structures. To identify and quantify the composition of individual components of a plant extract, high-performance liquid chromatography is the method of choice, ensuring rapid, selective, and reproducible qualitative and quantitative analysis of phenolic compounds. Recently, ultrahigh-performance liquid chromatography (UHPLC) combined with mass spectrophotometry has been introduced for the analysis of biologically active compounds in biological matrices. Such a combination and use of these methods allows for a fast and effective qualitative and quantitative analysis of different groups of phenolic compounds.

The paper presents the results of the investigation on the variability of the quantitative and qualitative composition of phenolic compounds in fruit samples of the species of *Rosa* L. grown in Lithuania: *Rosa rugosa* Thunb., *Rosa pimpinellifolia* L., *Rosa multiflora* Thunb., and *Rosa canina* L. and their cultivars. We found no publications that focus on the composition of phenolic compounds and antioxidant activity of *Rosa rugosa* Thunb. (cultivars “Rudolf”, “Dart’s Defender”, “Marie Bugnet”, “Fru Dagmar Hastrup”, and “Adam Chodun”), *Rosa multiflora* Thunb. (cultivar “Nana”), and *Rosa pimpinellifolia* L. (cultivars “Papula” and “Single Cherry”), except for *Rosa rugosa* Thunb. cultivar “Kornik”, grown in Lithuanian climatic conditions. The obtained results of the study will also allow for using the fruit of other cultivated *Rosa* L. species and their cultivars for the preparation of raw medicinal plant materials and for expanding the range of botanical raw materials for the development of food additives, for use in the pharmaceutical and food industries, respectively [16].

## 2. Materials and Methods

### 2.1. Plant Material

The fruit samples of *Rosa rugosa* Thunb. (cultivars “Rudolf”, “Dart’s Defender”, “Marie Bugnet”, “Fru Dagmar Hastrup”, “Kornik”, and “Adam Chodun”), *Rosa multiflora* Thunb. (cultivar “Nana”), *Rosa pimpinellifolia* L. (cultivars “Papula” and “Single Cherry”), and *Rosa canina* L. were obtained from the collection of Vilnius University Botanical Garden (54°40′56.762″, 25°14′52.832″ (World Geodetic System)).

### 2.2. Chemicals

The reagents used in the assays met all quality requirements and were of analytical grade. The following reagents were used in the study: ethanol 96% (*v/v*) (manufactured by SC Vilniaus degtinė, Vilnius, Lithuania), Folin–Ciocalteu reagent, gallic acid monohydrate, ferric chloride hexahydrate (FeCl_3_ × 6H_2_O), sodium acetate trihydrate (CH_3_COONa × 3H_2_O), DPPH (2,2-diphenyl-1-picrylhydrazyl), Trolox ((±) -6-hydroxy-2,5,7,8-tetramethylchromano-2-carboxylic acid), hexamethylenetetramine, chlorogenic acid, caffeic acid, phloridzin, quercetin, quercitrin, kaempferol-3-O-glycoside (+)-catechin, (−)-epicatechin, and (−)-epicatechin gallate (Sigma-Aldrich, Steinheim, Germany), sodium carbonate (Na_2_CO_3_), rutin (Carl Roth GmbH, Karlsruhe, Germany), glacial acetic acid 99.8% (Lachner, Neratovice, Czech Republic), 2,4,6-tripyridyl-s-triazine (TPTZ) (Alfa Aesar, Karlsruhe, Germany), concentrated hydrochloric acid, aluminum chloride hexahydrate (Fluka-Chemie, Buchs, Switzerland), sodium molybdate, sodium nitrite, and sodium hydroxide (Chempur, Tarnowskie Gory, Poland). Purified water was prepared, using the Milli-Q^®^ water-purification system (Millipore Co., Bedford, MA, USA).

### 2.3. Preparation of Rosa L. Fruit Samples

The *Rosa* L. fruits were dried in a well-ventilated and dry room. The dried *Rosa* L. fruit samples were ground with an Retsch GM 200 electric grinder. The ground raw material was stored in a dark and dry place, in tightly closed containers. The loss upon the drying of the raw material was determined by applying the technique described in the European Pharmacopoeia 07/2019:20232 [26].

### 2.4. Preparation of Rosa L. Fruit Extracts

During the study, 0.5 g (exact weight, weighed on a Sartorius CP64-0CE analytical balance (Sartorius AG, Gottingen, Germany)) of dried *Rosa* L. fruit powder was used, adding 10 mL of 40% (*v/v*) ethanol, and extracting in an ultrasonic bath Bandelin Sonorex Digital 10 P (Sigma-Aldrich, Darmstadt, Germany), for 50 min, at a temperature of 25 °C, at a frequency of 80 kHz, and at a power level of 1130 W. The obtained extract was filtered, and the dried *Rosa* L. fruit-powder mass remaining on the filter was washed with 40% (*v/v*) ethanol. The filtered extract was poured into 10 mL measuring flasks, adding 40% (*v/v*) ethanol up to the marking. Prior to the UHPLC analysis, the extracts were filtered through Carl Roth membrane filters (Carl Roth GmbH & Co. KG, Karlsruhe, Germany) with 0.22 µm pore size.

### 2.5. Spectrophotometric Assays

#### 2.5.1. Determination of the Total Amounts of Phenolic Compounds, Flavonoids, and Hydroxycinnamic Acid Derivatives

All spectrophotometric studies were performed on a UV–visible light (UV–Vis) spectrophotometer M550 (Spectronic CamSpec, Garforth, UK). The total phenolic content in the ethanol extracts of *Rosa* L. fruit was determined by using the Folin–Ciocalteu method [27], calculated from a gallic acid calibration curve, and expressed as mg gallic acid equivalent (GAE) per one gram of absolutely dry weight (DW) (mg GAE/g DW). The total amount of flavonoids in the ethanol extracts of *Rosa* L. fruit was determined by using the described methodology [28], calculated from a rutin calibration curve, and expressed as mg rutin equivalent (RE) per one gram of absolutely dry weight (DW) (mg RE/g DW). The total amount hydroxycinnamic acid derivatives in the ethanol extracts of *Rosa* L. fruit was determined by using the described methodology [29], calculated from a chlorogenic acid calibration curve, and expressed as mg chlorogenic acid equivalent (CAE) per one gram of absolutely dry weight (DW) (mg CAE/g DW).

#### 2.5.2. Evaluation of Antioxidant Activity

*Calculation of Antioxidant Activity of the Ethanol Extract of Rosa L. Fruit.* The antioxidant activity of the extracts was calculated from the Trolox calibration curve and was expressed as μmol of the Trolox equivalent (TE) per one gram of absolutely dry weight (DW).

*DPPH*^•^*Free Radical Scavenging Assay*. The DPPH^•^ free radical scavenging activity was determined, using the method proposed by Brand-Williams et al. [30]. DPPH^•^ solution in 96.3% *v/v* ethanol (3 mL, 6 × 10^−5^ M) was mixed with 10 μL of the ethanol extract of *Rosa* L. fruit. A decrease in absorbance was determined at a wavelength of 515 nm after keeping the samples for 30 min in the dark.

*FRAP Assay*. FRAP solution included TPTZ (0.01 M dissolved in 0.04 M HCl), FeCl_3_ × 6H_2_O (0.02 M in water), and acetate buffer (0.3 M, pH 3.6) (ratio 1:1:10). During the evaluation, 3 mL of a freshly prepared FRAP reagent was mixed with 10 μL of the extracts. An increase in absorbance was recorded at λ = 593 nm [31].

### 2.6. Chromatographic Assay

The variability of the qualitative and quantitative composition and content of phenolic compounds in *Rosa* L. fruit samples was evaluated, using ultrahigh-performance liquid chromatography–mass spectrometry, by applying the technique described and validated by Gonzalez–Burgos et al. [32]. Separation of phenolic compounds was performed with Acquity H-class UPLC system (Waters, Milford, MA, USA) equipped with Xevo triple quadrupole tandem mass spectrometer (Waters, Milford, MA, USA) with an electrospray ionization source (ESI), to obtain MS/MS data. YMC Triart C18 (100 × 2.0 mm; 1.9 μm) column (YMC Europe Gmbh, Dislanken, Germany) was used for analysis. Column temperature was maintained at 40 °C. Gradient elution was performed with mobile phase consisting of 0.1% formic acid water solution (solvent A) and acetonitrile (solvent B) with flow rate set to 0.5 mL min^−1^. Linear gradient profile was applied as follows for solvent A: initially 95% for 1 min, to 70% over 4 min, 50% over 7 min, and 95% over 2 min. Negative electrospray ionization was applied for analysis: capillary voltage, −2 kV; source temperature, 150 °C; desolvation temperature, 400 °C; desolvation gas flow, 700 L h^−1^; and cone gas flow, 20 L h^−1^. Collision energy and cone voltage were optimized for each compound separately. Mass spectrometry parameters for the analysis of phenolic compounds are presented in Table 1.

### 2.7. Statistical Analysis

Data analysis was carried out, using computer software Microsoft Excel 2016 (Microsoft Corp., Redmond, WA, USA) and SPSS Statistics 20 (IBM, Armonk, NY, USA). During the analysis, we calculated arithmetic means and standard deviations of three repeated measurements. A univariate dispersion analysis model (ANOVA) was applied for determining whether the differences between the compared data were statistically significant. Differences between the samples were determined by applying Tukey’s multiple comparison test. The correlation was evaluated by Pearson’s analysis. Differences at *p* < 0.05 were considered to be statistically significant. According to the quantitative composition of identified compounds, the tested samples were compared by the method of hierarchical cluster analysis, using squared Euclidean distances. Principal component analysis was performed, taking into account factors with eigenvalues higher than 1.

## 3. Results and Discussion

### 3.1. Determination of the Total Content of Phenolic Compounds, Flavonoids, and Hydroxycinnamic Acid Derivatives in Rosa L. Fruit Samples

Spectrophotometry is often used to assess the quality of raw medicinal plant materials and preparations made from them. The results obtained by applying this technique allow for determining the quantitative composition of groups of biologically active compounds. To evaluate the variability of phenolic compounds, flavonoids, and hydroxycinnamic acid derivatives in fruit samples of different *Rosa* L. species and cultivars, we selected the methodologies used for studies of raw medicinal plant materials.

**Figure 1:**
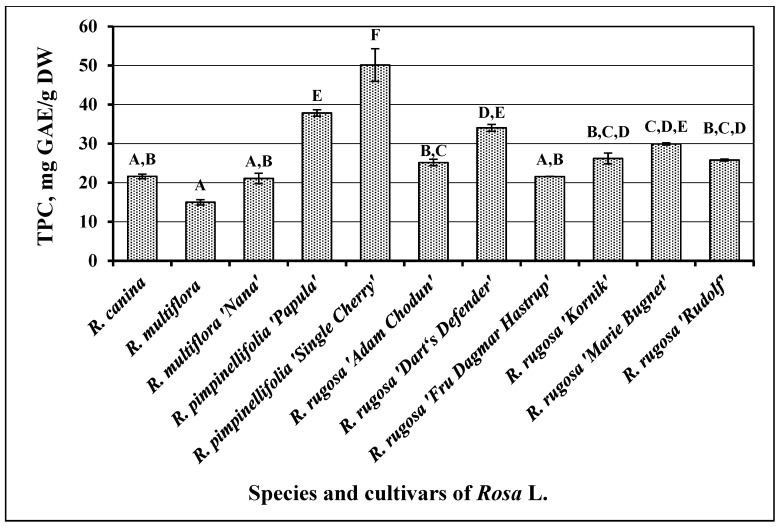
Variability of the total content of phenolic compounds in *Rosa* L. fruit samples; different letters indicate statistically significant (*p* < 0.05) differences between the samples.

The total amount of phenolic compounds in *Rosa* L. fruit samples was determined to vary from 14.99 ± 0.68 mg GAE/g to 50.13 ± 4.17 mg GAE/g (Figure 1). The mean total phenolic content in *Rosa* L. fruit samples was estimated to be 28.03 ± 2.05 mg GAE/g. The highest amount of phenolic compounds (50.13 ± 4.17 mg GAE/g, *p* < 0.05) was determined in fruit samples of *R. pimpinellifolia* cultivar “Single Cherry”. The lowest content of phenolic compounds was determined in fruit samples of *R. multiflora* (14.99 ± 0.68 mg GAE/g) and in samples of *R. multiflora* cultivar “Nana” (21.09 ± 1.31 mg GAE/g), as well as in fruit samples of *R. rugosa* cultivar “Fru Dagmar Hastrup” (21.57 ± 0.02 mg GAE/g) and in fruit samples of *R. canina* (21.61 ± 0.6 mg GAE/g) (Figure 1). There was no statistically significant difference in the quantitative composition of phenolic compounds between fruit samples of different *Rosa* L. species and cultivars (*p* > 0.05).

Demir et al. investigated the qualitative and quantitative composition of fruit samples of different species of *Rosa* L. [23]. The total amount of phenolic compounds determined in the samples studied by these scientists ranged from 31.08 to 52.94 mg GAE/g DW) [18]. Nađpal et al. analyzed fruit samples of *R. canina* and *Rosa arvensis* Huds. The total amount of the detected phenolic compounds ranged from 6.63 to 96.2 mg GAE/g DW [7].

Yang et al. investigated fruit samples of *Rosa roxburghii* Tratt. grown in China. Total content of phenolic compounds (173 mg GAE/g DW) in fruit samples of this species was significantly higher than contents which were determined in our study [33]. Such results may have been due to the different climatic conditions and interspecific differences between studied *Rosa* L. fruit samples.

**Figure 2:**
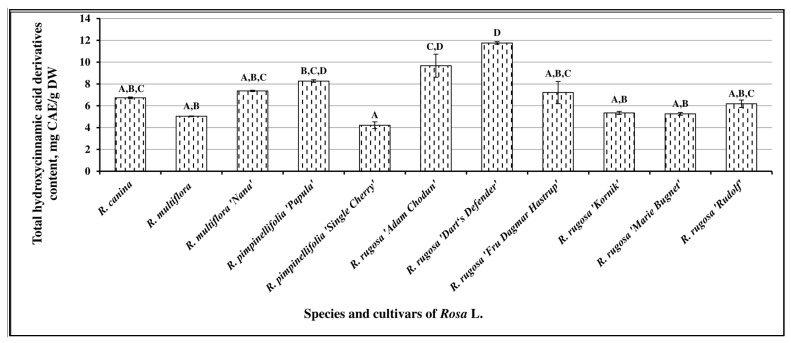
Variability of the total content of hydroxycinnamic acid derivatives in *Rosa* L. fruit samples; different letters indicate statistically significant (*p* < 0.05) differences between the samples.

The spectrophotometrically determined variability of the amount of hydroxycinnamic acid derivatives was from 4.22 ± 0.31 mg CAE/g to 11.76 ± 0.12 mg CAE/g (Figure 2). The mean total amount of hydroxycinnamic acid derivatives in *Rosa* L. fruit samples was determined to be 7.01 ± 0.49 mg CAE/g. The highest levels of hydroxycinnamic acid derivatives were determined in fruit samples of *R. rugosa* cultivar “Dart’s Defender” (11.76 ± 0.12 mg CAE/g) and *R. rugosa* cultivar “Adam Chodun” (9.68 ± 1.96 mg CAE/g). The lowest levels of hydroxycinnamic acid derivatives were determined in fruit samples of *R. pimpinellifolia* cultivar “Single Cherry” (4.22 ± 0.31 mg CAE/g), *R. multiflora* (5.04 ± 0.03 mg CAE/g), *R. rugosa* cultivar “Marie Bugnet” (5.26 ± 0.13 mg CAE/g), and *R. rugosa* cultivar “Kornik” (5.34 ± 0.14 mg CAE/g).

**Figure 3:**
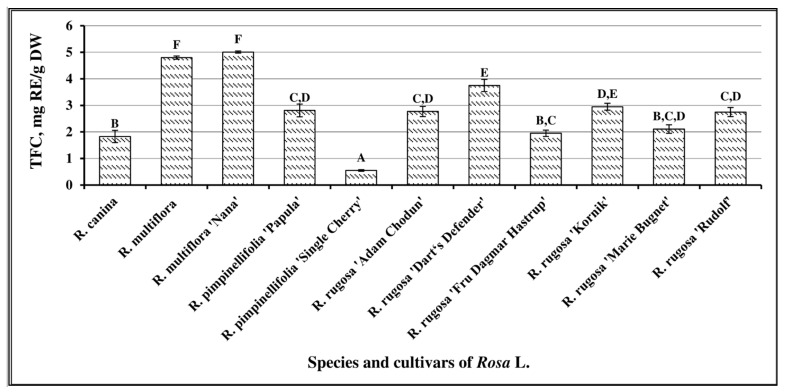
Variability of the total content of flavonoids in *Rosa* L. fruit samples; different letters indicate statistically significant (*p* < 0.05) differences between the samples.

Spectrophotometry showed that th total amount of flavonoids in the samples of different *Rosa* L. species and cultivars varied from 0.55 ± 0.03 mg RE/g to 5.01 ± 0.01 mg RE/g. The mean amount of flavonoids determined in *Rosa* L. fruit samples was 2.84 ± 0.27 mg RE/g. The highest total amount of flavonoids was determined in fruit samples of *R. multiflora* cultivar “Nana” (5.01 ± 0.01 mg RE/g) and *R. multiflora* (4.80 ± 0.06 mg RE/g). The lowest total amount of flavonoids (0.55 ± 0.03 mg RE/g, *p* < 0.05) was determined in fruit samples of *R. pimpinellifolia* cultivar “Single Cherry” (Figure 3). Nađpal et al. investigated the variability of the total flavonoid content in fruit samples of *R. canina* and *Rosa arvensis* Huds. Their total flavonoid content ranged from 0.63 to 1.48 mg RE/g [34]. Tahirovic et al. (2017) evaluated the total flavonoid content in fruit samples of *R. canina* and determined it to vary from 0.214 to 0.675 mg RE/g [35]. The comparison of the data of these studies with the results obtained in our study showed that fruit samples of *Rosa* L. grown in Lithuania had higher total amounts of flavonoids.

The data on the patterns of variability in the total phenolic and flavonoid content in *Rosa* L. fruit are scarce. Therefore, this study provides new knowledge about total phenolic and flavonoid content in *Rosa* L. fruit of the cultivars grown under Lithuanian climatic conditions; it also allows for the comparison of the obtained results with those of other studies and is valuable for carrying out a search for promising raw medicinal plant materials that accumulate biologically active substances.

### 3.2. Determination of the Qualitative and Quantitative Composition of Phenolic Compounds by UHPLC in Rosa L. Fruit Samples

**Figure 4:**
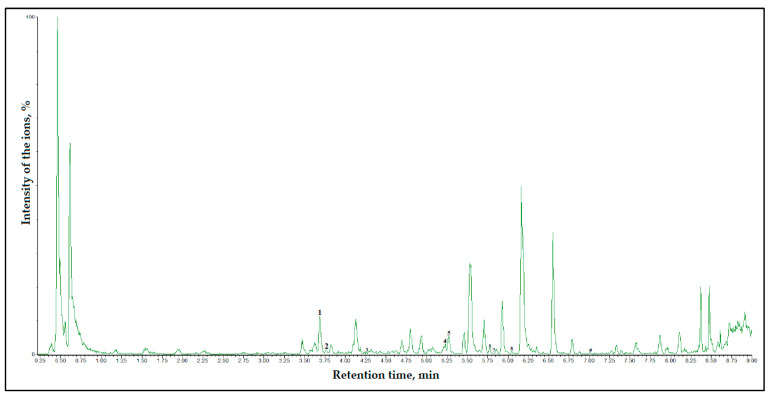
UHPLC chromatogram of fruit extract of *R. multiflora* (cultivar “Nana”). The numbers indicate the compounds identified: 1—(+)-catechin, 2—chlorogenic acid, 3—(−)-epicatechin, 4—rutin, 5—(−)-epicatechin gallate, 6—kaempferol-3-O-glucoside, 7—quercitrin, 8—phloridzin, and 9—quercetin.

In our study, we used UHPLC to analyze *Rosa* L. fruit samples. In the analyzed fruit samples, we identified phenolic acids (caffeic acid and chlorogenic acid) and flavonoids (quercetin, quercitrin, (+)-catechin, (−)-epicatechin, (−)-epicatechin gallate, rutin, phloridzin, and kaempferol-3-O-glucoside). The qualitative composition of phenolic compounds in the fruit extract of *R. multiflora* cultivar “Nana” determined via UHPLC is presented in the UHPLC chromatogram (Figure 4).

Qualitative analysis via UHPLC showed that the studied *Rosa* L. fruit samples contained phenolic acids—caffeic acid and chlorogenic acid. No phenolic acids were detected in fruit samples of *R. rugosa* cultivar “Adam Chodun” and *R. multiflora* (Table 2). Phenolic acids are often determined in organs of Angiospermae, they can act as co-compounds, and often have biological effects, determining the effects of medicinal preparations [36,37]. Phenolic acids have strong antioxidant [38], anti-inflammatory [39], renoprotective [40,41], hepatoprotective [42], and anti-diabetic [38,43] properties.

Using UHPLC, we analyzed the quantitative composition of the phenolic acids. The highest amount of caffeic acid (5.78 ± 0.07 µg/g, *p* < 0.05) was determined in fruit samples of *R. rugosa* cultivar “Rudolf”. The lowest amount of caffeic acid was detected in in fruit samples of *R. rugosa* cultivar “Fru Dagmar Hastrup” (3.46 ± 0.06 µg/g), *R. rugosa* cultivar “Dart’s Defender” (3.69 ± 0.07 µg/g), and *R. pimpinellifolia* cultivar “Single Cherry” (3.73 ± 0.03 µg/g) (Table 2). No caffeic acid was detected in fruit samples of *R. rugosa* cultivars “Adam Chodun” or “Marie Bugnet”, *R. canina*, *R. multiflora,* or *R. multiflora* cultivar “Nana”. The highest amount of chlorogenic acid (16.31 ± 0.85 µg/g, *p* <0.05) was determined in fruit samples of *R. multiflora* cultivar “Nana”. The lowest amount of chlorogenic acid was detected in fruit samples of *R. rugosa* cultivars “Dart’s Defender” (0.29 ± 0.01 µg/g) and “Marie Bugnet” (0.87 ± 0.01 µg/g) and *R. pimpinellifolia* cultivar “Papula” (1.62 ± 0.08 µg/g). No chlorogenic acid was detected in fruit samples of *R. rugosa* (cultivars “Adam Chodun”, “Kornik”, “Rudolf”, and “Fru Dagmar Hastrup”), *R. pimpinellifolia* cultivar “Single Cherry”, or *R. multiflora*.

The following compounds of the flavan-3-ol group were detected by using ultrahigh-performance liquid chromatography in *Rosa* L. fruit samples: (+)-catechin, (−)-epicatechin, and (−)-epicatechin gallate (Table 2). Flavan-3-ols are important for the human body as they exhibit antioxidant [44,45], anticancer [46], anti-inflammatory [47], platelet aggregation-modulating [48], and cholesterol-reducing [49,50] effects.

(+)-Catechin was identified in *Rosa* L. fruit samples of all the studied cultivars. Its content in *Rosa* L. fruit samples varied from 39.43 to 592.63 µg/g. The highest amount of (+)-catechin was determined in fruit samples of *R. multiflora* cultivar “Nana” (592.63 ± 6.39 µg/g, *p* < 0.05), and the lowest amount was determined in fruit samples of *R. pimpinellifolia* cultivar “Single Cherry” (39.43 ± 0.93 µg/g) and *R. rugosa* cultivars “Fru Dagmar Hastrup” (43.66 ± 0.86 µg/g), “Kornik” (50.38 ± 0.65 µg/g), and “Rudolf” (52.1 ± 0.99 µg/g). The highest amount of (−)-epicatechin (9.71 ± 0.04 µg/g, *p* < 0.05) was determined in fruit samples of *R. pimpinellifolia* cultivar “Papula”, and the lowest amount (0.02 ± 0.01 µg/g) *p* < 0.05)—in fruit samples of *R. rugosa* cultivar “Dart’s Defender” (Table 2). The amount of (−)-epicatechin gallate in *Rosa* L. fruit samples ranged from 79.6 to 149.29 µg/g. The highest amount of (−)-epicatechin gallate (149.29 ± 2.76 µg/g, *p* < 0.05) was determined in fruit samples of *R. pimpinellifolia* cultivar “Papula”, and the lowest amount—in fruit samples of *R. rugosa* cultivar “Marie Bugnet” (79.61 ± 0.83 µg/g), *R. multiflora* (84.32 ± 0.76 µg/g), and *R. rugosa* cultivar “Adam Chodun” (85.89 ± 1.53 µg/g). No (−)-epicatechin gallate was detected in fruit samples of *R. pimpinellifolia* cultivar “Single Cherry” or in *R. rugosa* cultivars “Rudolf” or “Fru Dagmar Hastrup” (Table 2).

Demir et al. studied the variability of the composition of phenolic compounds in the fruit of different species of *Rosa* L. In their studied fruit samples of *Rosa* L., (+)-catechin content ranged from 7.18 to 50.46 µg/g. Most of the *Rosa* L. fruit samples evaluated in our study contained higher amounts of this compound. Chlorogenic acid and (+)-catechin were identified in *R. canina* fruit samples grown in Poland [51]. (+)-Catechin and other compounds of flavan-3-ols group were identified in *R. canina* fruit samples, collected in Algeria [52]. Korean researchers identified chlorogenic and caffeic acids in fruit samples of *R. multiflora* [53]. Nađpal et al. examined fruit samples of *R. canina* and *R. arvensis* species. The (+)-catechin content (2.37–7.83 μg/g) reported by these researchers was significantly lower than the levels of this compound determined in our *Rosa* L. fruit samples, while the amount of (−)-epicatechin (1.72–4.74 μg/g) was close to the levels determined in our study. Demir et al. detected (−)-epicatechin gallate only in the fruit sample of *R. dumalis* subsp. *Boissieri*, and its content was lower than the amounts of this compound determined in the fruit samples of the cultivars evaluated in our study. The amounts of chlorogenic and caffeic acids determined by these scientists in their studied samples of various species of *Rosa* L. were higher than those determined in *Rosa* L. fruit samples evaluated in our study [18]. Such qualitative and quantitative differences in fruit composition may have been due to differences in *Rosa* L. species, different climatic conditions, soil composition, and other factors.

In the investigated extracts of *Rosa* L. fruit samples, flavonols were the most abundant group of phenolic compounds. These compounds are important for human health because of their strong antioxidant and anticancer activities [54,55,56]. The consumption of products rich in quercetin and its glycosides reduces the risk of cardiovascular [57,58] and neurodegenerative diseases [59,60].

Quantitative analysis of kaempferol-3-O-glucoside by ultrahigh performance liquid chromatography showed that fruit samples of *R. multiflora* cultivar “Nana” contained the highest amount of kaempferol-3-O-glucoside (46.47 ± 1.38 µg/g, *p* < 0.05), while the lowest amount of this compound was determined in fruit samples of *R. canina* (3.34 ± 0.51 µg/g) and *R. rugosa* variety “Rudolf” (0.68 ± 0.01 µg/g). The amounts of kaempferol-3-O-glucoside reported by Nađpal et al. (1.77 µg/g and 3.04 µg/g) were lower than the amounts of this compound determined in most of the *Rosa* L. fruit samples tested in our study [7]. The highest amount of phloridzin (28.75 ± 1.25 µg/g, *p* < 0.05) was determined in fruit samples of *R. multiflora* cultivar “Nana”, while the lowest amount of this compound was determined in fruit samples of *R. rugosa* cultivar “Rudolf” (1.51 ± 0.01 µg/g), *R. pimpinellifolia* cultivar “Papula” (3.99 ± 0.27 µg/g), and *R. rugosa* cultivar “Kornik” (5.73 ± 0.21 µg/g). The highest amount of quercetin (43.96 ± 0.12 µg/g, *p* < 0.05) was determined in fruit samples of *R. pimpinellifolia* cultivar “Single Cherry”. The lowest amounts of quercetin were determined in fruit samples of *R. multiflora* (5.56 ± 0.32 µg/g), *R. pimpinellifolia* cultivar “Papula” (6.73 ± 0.32 µg/g), and *R. multiflora* cultivar “Nana” (6.95 ± 0.06 µg/g). Quercitrin was identified in all *Rosa* L. fruit samples, its mean amount being 44.62 ± 18.32 µg/g. The highest amount of quercitrin (278.47 ± 2.65 µg/g, *p* < 0.05) was determined in fruit samples of *R. multiflora* cultivar “Nana”, and the lowest amount was determined in *R. rugosa* cultivars “Rudolf” (0.52 ± 0.42 µg/g) and “Adam Chodun” (1.75 ± 0.04 µg/g), *R. pimpinellifolia* L. cultivars “Single Cherry” (1.83 ± 0.04 µg/g) and “Papula” (2.52 ± 0.39 µg/g), *R. canina* (2.63 ± 0.01 µg/g), and *R. rugosa* cultivars “Fru Dagmar Hastrup” (2.79 ± 0.13 µg/g) and “Kornik” (3.83 ± 0.12 µg/g). The amount of quercitrin determined by Nađpal et al. varied from 27.1 to 113.0 µg/g [7]. The highest amount of rutin (19.44 ± 1.41 µg/g, *p* < 0.05) was determined in fruit samples of *R. multiflora* cultivar “Nana”, and the lowest amount was determined in *R. rugosa* cultivars “Kornik” (0.87 ± 0.11 µg/g) and “Adam Chodun” (1.54 ± 0.04 µg/g) (Table 3).

**Figure 5:**
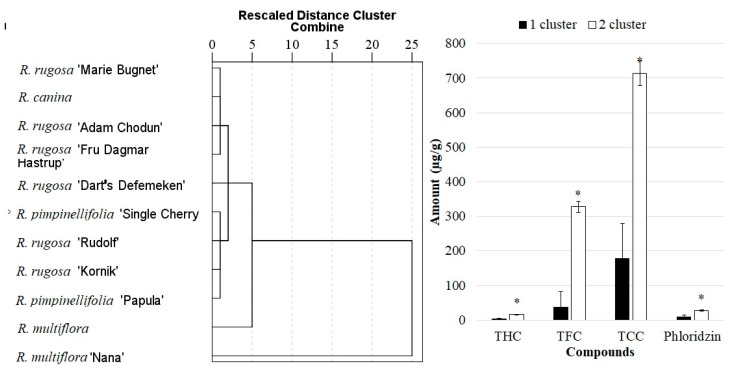
Dendrogram of the similarity of *Rosa* L. species, based on the composition of phenolic compounds in fruit samples. THC—total content of hydroxycinnamic acid; TCC—total content of flavan-3-ols; TFC—total content of flavonols. * Indicates statistically significant differences in the amounts of phenolic compounds (*p* < 0.05).

Hierarchical cluster analysis was performed for the samples of *Rosa* L. fruit, based on the content of dihydrochalcone phloridzin and the total content of flavan-3-ols, flavonols, and hydroxycinnamic acid. The investigated *Rosa* L. fruit samples have been grouped into two significant clusters (Figure 5). The first cluster consisted only of fruit samples of *R. multiflora* cultivar “Nana”, while the second cluster included all the other investigated *Rosa* L. fruit samples. By the total content, flavan-3-ols were the prevailing group of the identified biologically active compounds, followed by, in descending order, flavonols, hydroxycinnamic acid, and phloridzin. Fruit samples of *R. multiflora* cultivar “Nana” differed from the others, as they had the highest total contents of flavan-3-ols, flavonols, phloridzin, and hydroxycinnamic acid.

**Figure 6:**
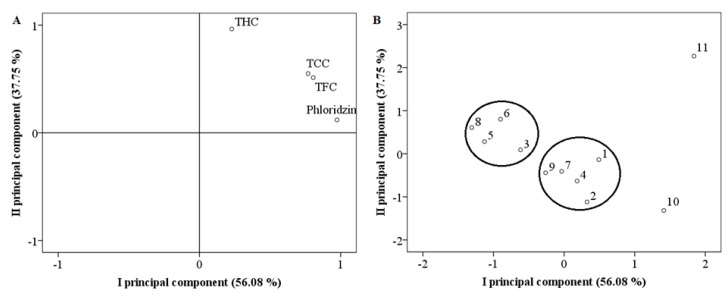
Principal component analysis: loading (**A**) and score (**B**) plots of fruit samples of different *Rosa* L. species. THC—total content of hydroxycinnamic acid; TCC—total content of flavan-3-ols; TFC—total content of flavonols. 1—*R. rugosa* cultivar “Dart’s Defender”; 2—*R. rugosa* cultivar “Adam Chodun”; 3—*R. rugosa* cultivar “Kornik”; 4—*R. rugosa* cultivar “Marie Bugnet”; 5—*R. pimpinellifolia* cultivar “Single Cherry”; 6—*R. pimpinellifolia* cultivar “Papula”; 7—*R. canina*; 8—*R. rugosa* cultivar “Rudolf”; 9—*R. rugosa* cultivar “Fru Dagmar Hastrup”; 10—*R. multiflora*; 11—*R. multiflora* cultivar “Nana”.

A principal component analysis of the identified biologically active compounds in *Rosa* L. fruit samples was performed (Figure 6). Two principal components explaining 93.83% of the total data variance were used for the in-depth analysis. The first principal component (PC I), which describes 56.08% of the total data variance, had a very strong positive correlation with the content of phloridzin (0.974) and a strong positive correlation with the total content of the other flavonoids (flavonols (0.806) and flavan-3-ols (0.769)). The second principal component (PC II), which describes 37.75% of the total data variance, had a very strong positive correlation with the total content of hydroxycinnamic acid (0.965). The clustering of the samples along PC I can be explained by the highest values of the total contents of flavonoids. Fruit samples of *R. multiflora* cultivars “Nana” and *R. multiflora* were distanced from all the others and were grouped at the positive side of the I PC as the total contents of flavonoids were high in these fruit samples. PC II differentiated these fruit samples by the total content of hydroxycinnamic acid. The highest total content of hydroxycinnamic acid scoring high in PC II was detected in the fruit samples of *R. multiflora* cultivar “Nana”. The other fruit samples were arranged into two distinct groups. Fruit samples of *R. pimpinellifolia* cultivar “Papula”, *R. pimpinellifolia* cultivar “Single Cherry”, *R. rugosa* cultivar “Kornik”, and *R. rugosa* cultivar “Rudolf” were scattering along negative PC I and positive PC II. In these fruit samples, the total contents of flavonoids were lower than the mean. Meanwhile, we determined that the total content of hydroxycinnamic acid in these fruit samples was higher than the mean. Fruit samples of *R. canina* and *R. rugosa* cultivars “Adam Chodun”, “Dart’s Defender”, “Fru Dagmar Hastrup”, and “Marie Bugnet” were located near the zero point of PC I. The content of phloridzin was higher than the mean. On the other hand, the total contents of flavonols and flavan-3-ols with high positive loadings in PC I were lower than the mean values. Moreover, fruit samples of these five species showed negative score values of PC II. The total content of hydroxycinnamic acid was determined in the range of the lowest to the mean values in these fruit samples.

As a consequence, *R. multiflora* cultivar “Nana” fruit samples formed a separate group indicating a different profile of phenolic compounds from other *Rosa* L. species. Meanwhile, our research revealed similarities between the other investigated fruit samples of *Rosa* L. species in the composition of phenolic compounds.

### 3.3. Determination of the Antioxidant Activity of Rosa L. Fruit-Sample Extracts In Vitro

The effectiveness of raw medicinal plant materials and preparations whose pharmacological activity is determined by phenolic compounds has been confirmed by abundant research data [61,62,63]. The use of raw medicinal plant materials for food and the use of phenolic compound-containing botanical preparations have been determined to have an association with the incidence of malignancies and cardiovascular and neurodegenerative diseases [64,65]. Epidemiological studies have demonstrated the ability of antioxidants to reduce or completely stop the progression of many chronic illnesses [66,67]. Studies on the antioxidant activity of phenolic compounds and their application for disease prevention have been carried out [68,69]. When conducting prospective studies on antioxidant activity, it is expedient to conduct an in vitro evaluation of the radical scavenging and reducing activity of the fruit-sample extracts of *Rosa* L. grown in Lithuanian collections. The results obtained during the study will be useful for the selection of *Rosa* L. cultivars in order to provide consumers with antioxidant-rich products, will help in the assessment and standardization of the quality of raw medicinal plant materials and their products, and will allow for predicting the antioxidant effect of *Rosa* L. fruit extracts in vivo.

**Figure 7:**
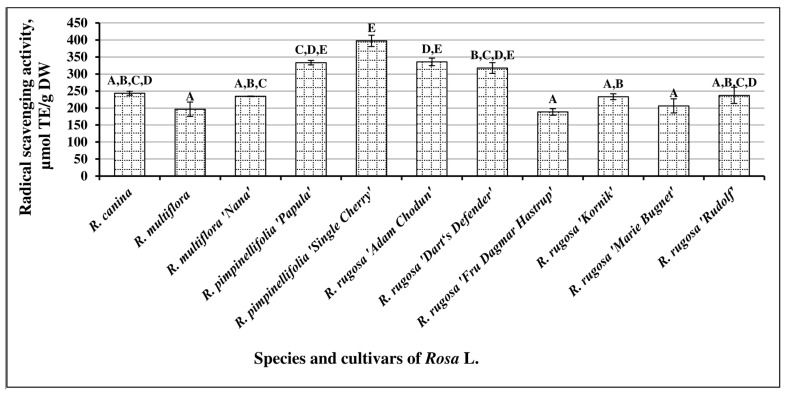
Variability of the radical scavenging activity of *Rosa* L. fruit-sample extracts in vitro; different letters indicate statistically significant (*p* < 0.05) differences between the samples.

Fruit-sample extracts of different species and cultivars of *Rosa* L. were analyzed, using an in vitro DPPH radical scavenging activity assay, and the variability of radical scavenging activity was determined to range between 188.39 ± 9.61 µmol TE/g and 397.2 ± 16.59 µmol TE/g (Figure 7). The mean radical scavenging activity of *Rosa* L. fruit samples in vitro was 265.68 ± 11.8 µmol TE/g. Extracts of fruit samples of *R. pimpinellifolia* cultivar “Single Cherry” and *R. rugosa* cultivar “Adam Chodun” demonstrated the strongest radical scavenging activity (respectively, 397.2 ± 16.59 µmol TE/g and 335.53 ± 11.13 µmol TE/g).

**Figure 8:**
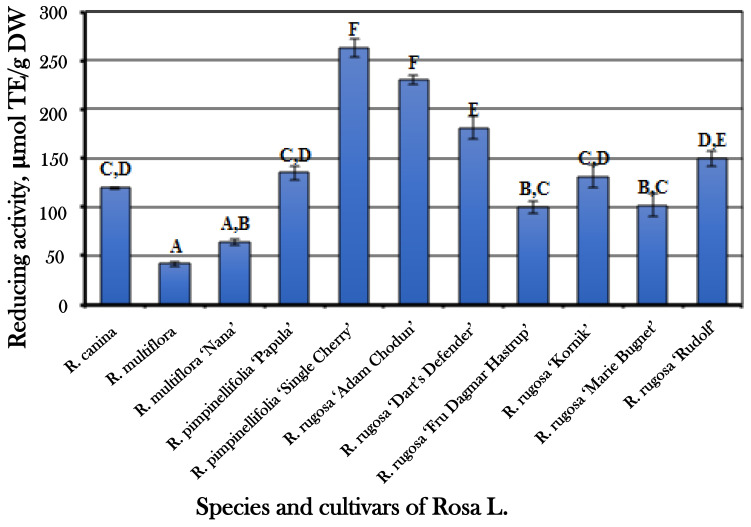
Variability of the reducing activity of *Rosa* L. fruit-sample extracts in vitro; different letters indicate statistically significant (*p* < 0.05) differences between the samples.

The evaluation of the reducing activity of *Rosa* L. fruit-sample extracts, using the in vitro FRAP assay, showed that the reducing activity ranged from 41.47 ± 2.3 µmol TE/g to 263.15 ± 9.83 µmol TE/g (Figure 8). The mean reducing activity in vitro was 137.81 ± 13.92 µmol TE/g. The strongest reducing activity was determined in the extracts of fruit samples of *R. pimpinellifolia* cultivar “Single Cherry” (263.15 ± 9.83 µmol TE/g) and *R. rugosa* cultivar “Adam Chodun” (229.56 ± 4.74 µmol TE/g).

There was a strong correlation between the total amount of phenolic compounds determined in fruit-sample extracts of different *Rosa* L. species and the radical scavenging and reducing activity of their extracts in vitro (R = 0.767 and 0.727, respectively, *p* < 0.05).

Polish researchers determined the in vitro antioxidant activity of fruit-sample extracts of plants of the genus *Rosa* L. by applying DPPH and FRAP assays (11.01 ± 0.74 mmol TE/100 g and 18.33 ± 0.71 mmol TE/100 g, respectively) [19]. In our study, extracts of *Rosa* L. fruit samples showed stronger radical scavenging activity in vitro, and their reducing activity was close to or even slightly higher than that determined by the aforementioned researchers. Tahirović et al. and Taneva et al., studied the reducing activity of *Rosa* L. fruit-sample extracts in vitro by applying the FRAP assay and obtained higher values than those we obtained in our study [35,70].

## 4. Conclusions

Scientific articles present research data on the qualitative and quantitative composition of biologically active compounds in *Rosa* L. fruit. Phenolic compounds determined in *Rosa* L. fruit have a wide range of biological effects and determine the use of *Rosa* L. fruit preparations in medical practice. To expand the provision of the raw material of *Rosa* L. fruit and to prepare high-quality raw medicinal plant materials, it is expedient to perform studies on the qualitative and quantitative composition of phenolic compounds in fruit of different cultivars of *R. rugosa* (“Rudolf”, “Dart’s Defender”, “Marie Bugnet”, “Fru Dagmar Hastrup”, “Kornik”, and “Adam Chodun”), *R. multiflora* (“Nana”), and *R. pimpinellifolia* (“Papula” and “Single Cherry”) grown in Lithuanian collections. In this study, we determined the overall variability of the quantitative composition of phenolic compounds, hydroxycinnamic acid derivatives, and flavonoids in *Rosa* L. fruit samples and evaluated the antioxidant activity of their extracts in vitro. The obtained results will allow for the selection of plant cultivars that accumulate the highest amounts of biologically active compounds and for the preparation of high-quality raw medicinal plant materials.

*Rosa* L. fruits are valuable raw medicinal plant materials that accumulate biologically active compounds—phenolic acids and flavonoids. Based on the obtained research data, we would recommend using fruit of *R. pimpinellifolia* cultivar “Single Cherry” and *R. rugosa* cultivar “Adam Chodun” for the production of various medicinal products. Extracts from the samples of the fruit of these *Rosa* L. species and cultivars showed the strongest antioxidant activity in vitro.

Hierarchical cluster analysis and principal component analysis revealed that *R. multiflora* “Nana” fruit samples had a different quantitative composition of phenolic compounds from the other species of the *Rosa* genus. Fruit samples of *R. multiflora* cultivar “Nana” had an exclusive phytochemical composition, as these fruit samples were determined to contain high amounts of all the identified phenolic compounds. This cultivar could be selected as a desirable raw material for the preparation of *Rosa* L. fruit products.

Recently, the raw material of the *Rosa* L. fruit has been used in the production of food, probiotic beverages, yogurts, and natural food additives. Biologically active compounds of *Rosa* L. fruit not only improve the quality but also have health-promoting and disease-preventive effects. The relationship between food and health is becoming increasingly important, as consumers want to eat healthy, tasty, and natural food grown in an organic environment. For this reason, it is important to investigate new *Rosa* L. species and cultivars, which could be a potential source of herbal raw material. Phenolic compounds are natural antioxidants having an impact on a prevention of various diseases. Our investigated fruit samples of *Rosa* L. species and cultivars could be characterized as having a broad variety of qualitative and quantitative composition of phenolic compounds. New knowledge about phytochemical composition, antioxidant activity and possibility to use *Rosa* L. herbal raw material in the production of functional food or as a perspective herbal raw material in practical medicine was obtained.

## Figures and Tables

**Table 1:** Mass spectrometry parameters for the analysis of phenolic compounds.

Compound	Parent Ion (m/z)	Daughter Ion (m/z)	Cone Voltage, V	Collision Energy, eV
**Caffeic acid**	179	107	36	22
**(−)-Epicatechin**	289	123	60	34
**(+)-Catechin**	289	123	60	34
**Quercetin**	301	151	48	20
**Chlorogenic acid**	353	191	32	14
**Phloridzin**	435	273	42	14
**(−** **)-Epicatechin gallate**	441	169	40	16
**Kaempferol-3-glucoside**	447	284	54	28
**Quercitrin**	447	300	50	26
**Rutin**	609	300	70	38

**Table 2:** Variability of the quantitative composition of phenolic acids and flavan-3-ols in *Rosa* L. fruit samples

Compound, µg/g DW	Caffeic Acid	Chlorogenic Acid	(+)-Catechin	(−)-Epicatechin	(−)-Epicatechin Gallate
*R. canina*	ND	2.68 ± 0.03 ^b^	107.93 ± 1.93 ^e^	ND	117.52 ± 2.27 ^b,c^
*R. multiflora*	ND	ND	145.37 ± 3.38 ^d^	ND	84.32 ± 0.76 ^d^
*R. multiflora* “Nana”	ND	16.31 ± 0.85 ^a^	592.63 ± 6.39 ^a^	2.74 ± 0.07 ^b^	126.15 ± 1.70 ^b^
*R. pimpinellifolia* “Papula”	4.81 ± 0.02 ^b^	1.62 ± 0.08 ^b,c^	89.17 ± 1.25 ^f^	9.71 ± 0.04 ^a^	149.29 ± 2.76 ^a^
*R. pimpinellifolia* “Single Cherry”	3.73 ± 0.03 ^c,d^	ND	39.43 ± 0.93 ^g^	0.38 ± 0.02 ^e^	ND
*R. rugosa* “Adam Chodun”	ND	ND	93.73 ± 1.83 ^e,f^	1.99 ± 0.04	85.89 ± 1.53 ^d^
*R. rugosa* “Dart’s Defender”	3.69±0.07 ^c,d^	0.29±0.01 ^c^	232.08 ± 3.92 ^b^	0.02 ± 0.001 ^f^	122.67 ± 3.41 ^b,c^
*R. rugosa* “Fru Dagmar Hastrup”	3.46 ± 0.06 ^d^	ND	43.66 ± 0.86 ^g^	0.90 ± 0.05 ^d^	ND
*R. rugosa* “Kornik”	3.95 ± 0.10 ^c^	ND	50.38 ± 0.65 ^g^	ND	113.01 ± 2.42 ^c^
*R. rugosa* “Marie Bugnet”	ND	0.87 ± 0.01 ^b,c^	197.96 ± 3.47 ^c^	0.22 ± 0.01 ^e,f^	79.61 ± 0.83 ^d^
*R. rugosa* “Rudolf”	5.78 ± 0.07 ^a^	ND	52.10 ± 0.99 ^g^	ND	ND

DW, dry weight; different letters indicate statistically significant differences in the amounts of individual compounds of these groups in *Rosa* L. fruit samples (*p* < 0.05). ND−Not detected.

**Table 3:** Variability of the quantitative composition of flavonols and phloridzin in *Rosa* L. fruit samples

Compound, µg/g DW	Kaempferol-3-O-glucoside	Phloridzin	Quercetin	Quercitrin	Rutin
***R. canina***	3.34 ± 0.51 ^e,f^	1.76 ± 1.08 ^c,d^	ND	2.63 ± 0.01 ^e^	ND
***R. multiflora***	10.14 ± 0.71 ^d^	20.78 ± 1.12 ^b^	5.56 ± 0.32 ^c^	142.58 ± 2.94 ^b^	6.51 ± 0.44 ^c^
***R. multiflora* “Nana”**	46.47 ± 1.38 ^a^	28.75 ± 1.25 ^a^	6.95 ± 0.06 ^b^	278.47 ± 2.65 ^a^	19.44 ± 1.41 ^a^
***R. pimpinellifolia* “Papula”**	ND	3.99 ± 0.27 ^f^	6.73 ± 0.32 ^b,c^	2.52 ± 0.39 ^e^	ND
***R. pimpinellifolia* “Single Cherry”**	ND	ND	43.96 ± 0.12 ^a^	1.83 ± 0.04 ^e^	ND
***R. rugosa* “Adam Chodun”**	12.47 ± 0.52 ^d^	13.26 ± 0.75 ^c,d^	ND	1.75 ± 0.04 ^e^	1.54 ± 0.04 ^d^
***R. rugosa* “Dart’s Defender”**	38.61 ± 0.84 ^b^	14.96 ± 0.94 ^c^	ND	32.09 ± 1.03 ^c^	9.97 ± 0.28 ^c^
***R. rugosa* “Fru Dagmar Hastrup”**	5.57 ± 0.43 ^e^	11.85 ± 0.25 ^c,d^	ND	2.79 ± 0.13 ^e^	ND
***R. rugosa* “Kornik”**	12.52 ± 0.56 ^d^	5.73 ± 0.21 ^e,f^	ND	3.83 ± 0.12 ^e^	0.87 ± 0.11 ^d^
***R. rugosa* “Marie Bugnet”**	22.62 ± 1.23 ^c^	9.55 ± 0.48 ^d,e^	ND	21.88 ± 0.70 ^d^	14.03 ± 0.46 ^b^
***R. rugosa* “Rudolf”**	0.68 ± 0.01 ^f^	1.51 ± 0.01 ^f^	ND	0.52 ± 0.03 ^e^	ND

Different letters indicate statistically significant differences in the amounts of individual compounds of these groups in *Rosa* L. fruit samples (*p* < 0.05). ND−Not detected.

## Data Availability

All datasets generated for this study are included in the article.
